# Use of Virtual Reality in Physical Therapy as an Intervention and Diagnostic Tool

**DOI:** 10.1155/2024/1122286

**Published:** 2024-01-25

**Authors:** Hamid Bateni, Jenna Carruthers, Rebecca Mohan, Seyedamirhossein Pishva

**Affiliations:** ^1^Physical Therapy Program, School of Allied Health and Communicative Disorders, Northern Illinois University, 1425 W. Lincoln Hwy., DeKalb, IL 60115, USA; ^2^College of Osteopathic Medicine, Kansas City University, 1750 Independence Ave, Kansas City, MO 64106, USA

## Abstract

Within the past decade, the integration of computer-generated virtual realities (VRs) has witnessed a significant rise in the field of healthcare, particularly in diagnosis and treatment applications. These VR systems have found extensive use in physical therapy, rehabilitation, research, and assessment. This narrative review article is aimed at providing a comprehensive overview of the literature regarding the implementation of VR in the physical therapy profession. The primary objective of this review is to provide information to clinicians about the diverse applications of VR and its potential advantages in intervening across various patient populations and diagnoses during rehabilitation therapy. Through in-depth discussions with experts and a thorough review of pertinent literature, several significant aspects of the topic were identified. Subsequently, we carried out an online search to investigate the prevalent utilization of VR systems within healthcare, both as assessment tools and for therapeutic interventions. Our examination encompassed a total of 56 articles, with supplementary references incorporated as required.

## 1. Introduction

Virtual reality (VR) refers to an advanced form of human-computer interface where operators enter and interact with a highly naturalistic computer-generated environment. Such interactions are primarily achieved via delivery of an optical illusion that provides visual information. Within the past decade, the use of computer-generated VRs is becoming more common in healthcare, both for the purpose of diagnosis and treatment. Along with other areas of healthcare, physical therapy practice is experiencing changes due to recent technologies. The physical therapy profession primarily utilizes patient assessments and goal-specific exercises for treatment [1]. Physical therapy strives to create a safe and replicable environment, where patients can improve upon limitations in specific activities of daily living (ADL) that resemble their day-to-day functioning. Previous attempts were made to explore monotonous physical therapy treatment, such as computer gaming systems [2, 3], particularly to benefit individuals who do not have access to therapists. Often recommended physical therapy treatment exercises can become monotonous proving difficult to motivate patients, whereas VR has been shown to improve motivation in patients [4–6]. In fact, VR through telerehabilitation offers remote therapy for patients who do not have the luxury to travel to urban-located clinics [4]. As a result, VR technology is becoming more popular for both assessment and intervention in the physical therapy profession [7]. VR is similar to conventional therapy because it offers a controlled environment within an augmented environment while influencing and tracking patient's kinematic responses [7, 8].

This narrative review attempts to summarize the literature on the use of VR in the physical therapy profession. The intention of this review is to inform clinicians on the utilization of different types of VR and the potential benefits VR can have on intervening in a variety of patient populations and diagnoses in rehabilitation therapy.

## 2. Methods

This paper has been developed by collecting information from a diverse range of sources. The authors' initial experiences, obtained through conducting studies using VR systems, served as a foundation for understanding VR technology. Additionally, valuable insights from experts were gathered through meetings and discussions, shaping the outline of this paper.

In pursuit of comprehensive coverage, each subheading of this paper was subjected to an extensive search on different databases including PubMed, Google Scholar, and CINAHL. Relevant articles were carefully selected and reviewed to support the content.

## 3. Results

**Figure 1:**
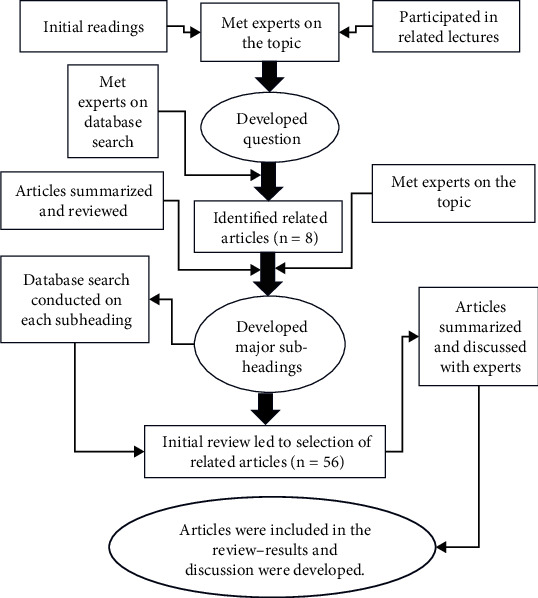
Flowchart of the development of question and process of selecting articles to be included in this narrative review. A total of 56 articles were selected and reported.

Through in-depth discussions with experts and a thorough examination of related literature, with a specific focus on the use of VR in physical therapy, several significant sections were identified and subsequently incorporated into this paper.  Figure 1 illustrates the progression of question formulation and article curation within this review. The initial article assessment process led to the identification of three distinct subcategories: the prevalent utilization of VR systems in healthcare, the utilization of VR for therapeutic interventions, and the application of VR in assessment procedures. A total of 56 articles were meticulously chosen and subjected to thorough evaluation. Furthermore, as the review unfolded, supplementary articles were included to provide comprehensive coverage. This paper offers a comprehensive discussion of all selected articles.

## 4. Discussion

### 4.1. Common VR Systems Used in Healthcare

VR is a concept that originated in the 1960s, but prototype VR technology started being introduced in the late 1980s [9]. VR technology offers a computer-generated environment that allows individuals to interact with a virtual or 3-dimensional (3D) environment [7, 10–12]. Not all computer-simulated virtual environments or virtual experiences are recognized as VR. In the 1960s, around the same time VR originated, augmented reality (AR) was another developing concept [9, 13]. AR offers a type of virtual experience, where simulated objects are introduced to the user's real-world environment to allow a user to interact and engage with virtual objects [9, 13–15]. AR-related technology encompasses headsets, handheld cameras, or, more recently, phone and tablet applications that project or overlay virtual images to a user's viewpoint of the real environment [13, 14, 16]. In contrast, VR technology is progressive, because it offers some level of immersion in a VR environment, such as a semi-immersive projector-based virtual environment that is displayed on a projector screen surrounding a user [7, 9, 17]. Recent innovative VR technology offers fully immersive VR environments that allow complete immersion for a user to interact with a virtual world, most commonly, through a head-mounted display or goggles [14, 16, 18]. When compared to AR, VR technology is primarily acknowledged and studied in healthcare to create virtual experiences that can transcend into rehabilitation care [7, 10, 19].

There are several different fully immersive VR system technologies. Examples of this type of VR include Oculus VR, CAREN, Samsung Gear VR, Psious, and Virtualis, which will be individually discussed further [20–24]. Fully immersive VR typically involves wearing a headset. Examples of augmented reality include Nintendo Wii variations, Kinect for Xbox 360, Reh@City, and BTS Nirvana VR [25–30].  Table 1 compares and contrasts common VR systems that are typically used either for assessment or for treatment in healthcare. Based on our research findings, the most common VR systems that have been used in healthcare are Oculus and Nintendo Wii. Developers often use these systems to develop their own assessment or training modules.

Oculus VR consists of two types of headsets, the Quest 2 and Rift S with built-in 3D positional audio and 6 degrees of freedom to track the movement of the head and body [31]. Quest 2 is an advanced portable “all-in-one VR headset,” which requires an Oculus headset and hand touch controllers that connect with a smartphone application or gaming computer [31]. In contrast, Rift S provides a higher resolution experience requiring a headset and Oculus hand touch controllers along with a VR-compatible computer for connectivity [31, 32].

A more advanced type of VR used in research and clinical settings is CAREN (computer-assisted rehabilitation environment) developed by Motek Medical [33, 34]. Equipment includes a real-time motion-capturing system and a motion base consisting of force plates and a treadmill [33–35]. This dynamic platform provides 6 degrees of freedom to track total head and body movement within a fully immersive VR experience for the purpose of improving gait and stability [33–35]. The patient is safely secured within a harness as gait and stability are challenged in the VR experience [34]. The VR environment can be projected within a 360-degree theater system or a projector system with surrounding audio [34, 35].

One popular VR system that is powered by Oculus is Samsung Gear VR. This system is designed to turn your cell phone into a portable VR system [31]. Individuals can choose between various apps on their phone and then connect it to their Samsung Gear headset to play the game in a VR environment [31]. Benefits of this headset include it weighing only 345 grams, which makes it lightweight, and it is designed for a comfortable fit for the participant [36]. In addition, Samsung VR has a 101-degree field of vision which gives the person a decent size field to immerse themselves in [36]. The system is relatively inexpensive for the headset and controller, so it would be easily accessible for places interested in using VR with their patients [31].

Psious also introduced an “all-in-one VR platform” with a headset, biofeedback sensor, and compatible VR therapy environments accessible through the Psious platform [37]. Psious focuses on offering VR-incorporated therapeutic intervention for mental illnesses, which can be utilized by mental health professionals [37]. Psious offers mental health professionals to access and configure a variety of environmental stimulus, that can be difficult to replicate in the clinic, such as airplane take-offs and elevator usage, while maintaining a controlled virtual environment with the VR control systems [37].

Virtualis is another VR system that was designed by a healthcare professional and a research team [38]. Applications with this system can be used for patients with a variety of conditions to work on ADLs, balance, motor function, hemineglect, cognition, proprioception, and mirror therapy in a virtual environment [38]. The system has specific assessment and rehabilitation options for individuals with balance and vestibular disorders [38]. Virtualis has been used in 450 facilities around the world and about 25 facilities in the United States alone [38]. Some benefits of Virtualis include its user-friendly interface, and it was developed by a healthcare professional specifically to be used in a healthcare environment [38].

The 2006 Nintendo Wii handheld wireless controller introduced a VR experience through a player's avatar [26]. The handheld device encompasses hand and arm motion sensors, which detects changes in direction and speed, while a 2-point infrared light sensor captures the overall player's motion to display on a television screen [26]. The benefit of the Nintendo Wii is that it offers accessories that can be utilized in the rehabilitation setting. These accessories enhance the VR system by including sensory-detecting remote controls, racing wheels, balance boards, nunchuk remote accessories, and sensor bars [39]. This VR system was marketed towards any consumer interested in its simplicity and at-home user-friendliness [40–42]. This suggests the Nintendo Wii to be one of the popular VR devices being mentioned several times in the rehabilitation setting, as discussed in the studies in this review.

Xbox developed the Kinect system to allow users to interact with the games they are playing. The Kinect system works with an Xbox 360 or an Xbox One with an adapter to create an immersive experience where individuals can use their body to control their game [43]. One benefit of this system is that the Kinect sensor itself is very inexpensive, but individuals will also need to purchase an Xbox if they do not have one. This system would be an inexpensive option for healthcare settings to have at their disposal.

A unique VR system is Reh@City, which is a simulated environment for individuals to navigate a city and engage in ADLs [29]. While using Reh@City, participants perform ADLs in common places in the community, including the supermarket, the bank, the pharmacy, and the post office [29]. This system is beneficial because it allows individuals to practice important real-world tasks in a realistic simulated environment. Since the system was designed with older individuals in mind, the system is easy to navigate, and the environment includes simple building shapes, so participants can better memorize their routes in the simulated community [29]. This system can be run on a desktop computer with a joystick controller [29]. Since Reh@City was developed for research studies, it may be difficult to obtain for use in healthcare settings.

The BTS Nirvana system was developed specifically for the rehabilitation of individuals with neurological disorders [44]. This system works by projecting different scenarios onto the floor or walls to allow the patient to react to the given stimulus [44]. According to the Nirvana website, over 200 rehabilitation settings are using Nirvana today [44]. Benefits of this system include having six different games to choose from; being compatible with a variety of different devices, including cell phones, tablets, and computers; and providing a report on the patient's performance to assess strengths and weaknesses [44].

### 4.2. Use of VR for Assessment and Diagnosis

VR has been used as a tool in diagnosing certain health conditions. The two conditions that have been reported in literature, where VR is used as a diagnostic tool, are postural stability in patients with Alzheimer's disease and those with balance impairments secondary to a mild traumatic brain injury (mTBI). Wright et al. [45] examined a portable VR system and explored to see if it could be used in place of a sensory organization test (SOT). The experiment included sixty-seven physically active college students (56 healthy, 11 concussed) who performed six standing postural tasks with both the SOT and VR systems [45]. The results indicated that patients with a mTBI perform worse than the healthy participants which demonstrated the VR system's ability to detect balance impairments in individuals following a mTBI [45]. The study conducted by Gago et al. [46] studied the ability to perform compensatory postural adjustments in a group of individuals with Alzheimer's disease versus healthy individuals. The study included twenty-one individuals with Alzheimer's disease and nineteen healthy individuals [46]. The experiment involved the use of low-frequency and high-frequency bands to look at compensatory postural adjustments [46]. The results reported that individuals with Alzheimer's disease who had a history of falls demonstrated a deficit in their postural stability as compared to healthy individuals [46]. These two studies indicated that VR can be used as a tool in the process of diagnosing impairments in balance and postural stability.

#### 4.2.1. Gait and Postural Balance

VR has been used to assess gait in the physical therapy setting. One study by Matar et al. [47] used VR to investigate the gait differences in individuals with Parkinson's disease with and without freezing gait. The results of the study showed that individuals with freezing gait have increased stepping latencies in response to stimuli and increased motor latencies in response to environmental factors, such as narrow doorways and opening a sliding door, compared to individuals without freezing gait [47].

Balance in patients with subacute mTBI has been assessed using VR [45]. The study included healthy patients and patients who were subacute mTBI [45]. The patients underwent balance testing using the VR system and the sensory organization test (SOT) [45]. The study participants with a mTBI performed worse more deficits than the healthy participants [45]. The results of this study demonstrate that VR can be used in rehabilitation by accurately detecting balance impairments in patients with a mTBI [45].

Compensatory postural adjustments in individuals with Alzheimer's disease have been studied using VR [46]. The individuals with Alzheimer's disease demonstrated the worst postural stability and a time lag in their reaction to the high-frequency band [46]. This study demonstrates that VR can be used to determine if an individual with Alzheimer's disease is a fall risk by testing their postural stability and lag time in reacting to provoked postural adjustments [46].

### 4.3. Use of VR for Intervention

In addition to serving as a diagnostic tool, VR has also been used for intervention in older adult population and other populations to address functional deficits. These functional deficits encompass gait performance, balance deficits, postural sway, upper extremity functional reach and grasp, endurance, or phantom limb pain.

#### 4.3.1. Gait

Several researchers have studied the use of VR incorporation for motor-cognition sequencing with gait and locomotion. Phu et al. [48] reported improved gait speed with the intervention group that utilized the Wii Fit for balance rehabilitation amongst older adults at risk of falls [48]. Some researchers found no significant results with VR usage to improve gait and locomotion [48]. For instance, it is unsure whether VR can improve gait speed, since Gandolfi et al. utilized telerehabilitation and VR to compare balance training and in-clinic balance training along with secondary outcome measures that found no clinically significant differences with 10-meter walking speed in a population with Parkinson's disease [49]. However, Ferraz et al. found that the group of older adults with PD receiving intervention with Kinect Adventures VR showed significant improvement with 6 MWT and 10MWT [50], thus demonstrating improvement with gait speed and endurance [50]. Similarly, patients with stroke who received VR-based treatment demonstrated significantly improved gait speed, with respect to 10MWT [51, 52]. Additionally, Feng et al. did find significant results with improving gait while negotiating obstacles, with respect to the Functional Gait Assessment, in patients with PD receiving the VR intervention. Robinson et al. [53] utilized Nintendo Wii to assess the progress of balance and gait in patients with multiple sclerosis but saw no improvements with gait [54]. Future studies might find otherwise, such as Mirelman et al., proposing a protocol design for a study using Kinect motion sensor and computer simulations to create a virtual obstacle course [55]. In this proposed study, older adults walk on a treadmill to potentially decrease falls over 6 months postintervention and improve gait speed for 1-minute walking under 3 conditions consisting of comfortable walking speed, negotiating obstacles, or dual tasking [55]. Related research should be conducted to further see if there is more evidence to support VR inclusively improving gait, though it is suggested that VR intervention does impact balance and walking capacity. These discussed studies all have limitations that need to be considered. For instance, some studies had small sample sizes and no long-term follow-up [50–52]. Mirelman et al., on the other hand, had participants' self-report falls which could potentially be a source of bias [55]. As such, the generalizability of these results may be limited, particularly because patient participants present on a continuum and some may respond better to VR than others.

#### 4.3.2. Balance

VR has been used as an intervention to improve balance deficits in various populations. For example, older adults and patients with PD, stroke, or multiple sclerosis (MS) intervened with a type of VR technology demonstrating improved balance most commonly seen with improved scores with the Berg Balance Scale (BBS) as a primary outcome measure [28, 30, 49, 52, 54, 56–65]. The Berg Balance Scale is a 14-item performance outcome measure to assess balance in older adults, specifically ages 65 and over [66, 67]. Yesilyaprak et al. found older adults with a history of falls to benefit from VR-based standing balance exercises using BTS Nirvana reporting significant changes on a 95% confidence interval with a 3.4 point change on the BBS from baseline to post-training [30]. Lee et al. [61] assessed patients with PD intervened with VR K-Pop Dance Festival using Nintendo Wii and reported significantly improved BBS scores (46.0 ± 1.3 to 48.1 ± 3.0; *p* < 0.05) unlike the control group at preintervention and postintervention time periods. Gandolfi et al. [49] also assessed patients with PD with *TeleWii* balance training programing with Nintendo Wii exergames compared to conventional sensory integration balance training and found significant differences within both groups from preintervention and postintervention scores and from postintervention to follow-up evaluation on the BBS. There were also significant in-between group differences for BBS scores from baseline to postintervention at 7 weeks reporting a *p* value of 0.04 [49]. Severiano et al. [59] conducted an observational study on PD patients intervened with VR games using Nintendo Wii, Wii-Remote, and Wii Balance Board, and comparative results from preintervention to postintervention were significant for the tightrope walk and Ski Slalom VR games, and correlation results between the Ski Slalom VR game and BBS were significant at postintervention, in respect to Spearman's correlation test [59]. This suggests that certain VR games can be effective for balance training in the PD population after 20 treatment sessions [59]. Ozgonenel et al. [60] studied the effects of Xbox games on PD patients and found postintervention scores to be significantly better for the intervention group reported as 53 ± 4 compared to the control group 47 ± 8 (*p* = 0.004) from preintervention to postintervention on the BBS [60]. Cho et al. explored the effects of VR balance training using a Nintendo Wii Fit balance board in stroke patients and reported that after 6 weeks of intervention, this group demonstrated significant improvement in BBS scores (39.09 to 43.09, *p* value < 0.05), and so did the control group, but in-between group comparisons show significant changes in BBS and TUG in the VR group, in respect to *p* value < 0.05 [64]. In another study, Cho et al. [63] studied the effects of a Virtual Walking Training program using a projector screen in front of a treadmill and found stroke patients to have significantly improved on BBS with scores improving from baseline to postintervention (36.71 ± 2.28 to 40.85 ± 1.67 scores, *p* value < 0.05), along with the control group, but overall, the VR group showed significantly greater improvement in-between group scores on the BBS. In et al. [62] studied the effects of VR reflection therapy in patients with chronic stroke and found significant changes in BBS scores between the VR group (45.46 ± 4.12 to 49.08 ± 2.72) and the control group (44.75 ± 3.02 to 46.08 ± 2.97) postintervention within groups, and in-between group differences were significant and favored towards the VR group, in respect to a *p* value < 0.05. Lee et al. [65] studied augmented reality postural control training using a computer-mounted camera and super video graphics array head-mounted display on chronic stroke patients, and *post hoc* analysis found significant results improvement on BBS for the intervention group (*p* = 0.007). Lloréns et al. [52] studied the effects of VR-based stepping exercise to a control group, and the results showed significant improvement for both groups on the BBS with the VR group showing significantly more difference on 95% CI (1.9 to 5.6, *p* value < 0.05) compared to the control group (0.8 to 2.8). Lozano-Quilis et al. [56] studied the effects of a Kinect-based VR system, RemoviEM, on patients with MS that encompassed motor games TouchBall, TakeBall, and StepBall and reported significant time (*p* = 0.014) and group by time (*p* = 0.030) interaction in BBS scores [56]. Results for SLB were also reported as significant time effect and group-by-time interaction for the right foot from preintervention to postintervention scores [56]. Gutierrez and Galan Del Rio [28] also studied the effects of VR using the Xbox360® console with Microsoft® Kinect to play throwing, hitting, and dodging games while taking the form of a virtual avatar in patients with MS and reported significant in-between group differences for the VR group on the BBS and Tinetti balance assessment score (*p* value < 0.001). Gutierrez and Galan Del Rio [28] concluded that VR should be considered as an alternative, if not a better approach, for balance intervention for the MS populations above.

Specifically, the older adult population and Parkinson's disease (PD) population demonstrated improved scores with the Timed Up and Go (TUG) as a primary outcome assessment after receiving VR intervention [30, 48, 54, 58, 60–64, 68]. The TUG is a valid test that assesses mobility and fall risk in older adults with or without an assistive device for a distance of 3 meters or 10 feet [69, 70]. Researchers found that TUG scores significantly improved with either VR intervention or conventional exercise programs [30, 48, 68]. These researchers support VR intervention as an alternative treatment route to improve outcome measures related to balance training in older adults [30, 48, 68]. Yesilyaprak et al. [30] reported that older adults with a history of falls benefit both from VR-based standing balance exercises using BTS Nirvana and conventional balance exercises with significant changes and scores not indicative of fall risk for similar aged groups on the TUG from baseline to postintervention. Ozgonenel et al. [60] studied the effects of Xbox games on PD patients and also found postintervention scores to be significantly better for the intervention group (reported as 11 ± 4 compared to the control group 20 ± 8, *p* = 0.001) from preintervention to postintervention on the TUG. Cho et al. [64] also found stroke patients receiving VR balance training using a Nintendo Wii Fit balance board to show significant improvement on TUG scores (21.74 seconds to 20.40 seconds, *p* value < 0.05), and so did the control group with in-between group comparisons showing significant changes in BBS and TUG in the VR group, in respect to *p* value < 0.05 [64]. In another study, Cho et al. [63] explored the effects of the Virtual Walking Training program and found stroke patients to have significantly improved on the TUG from baseline to postintervention (22.13 ± 5.82 to 21.18 ± 5.86 seconds, *p* value < 0.05), along with the control group, but overall, the VR group showed significantly greater improvement in-between group times on the TUG. Lee et al. [65] who studied augmented reality postural control training on chronic stroke patients reported *post hoc* analysis results to be significant for improvement on the TUG for both the intervention group (*p* = 0.007) and the control group (*p* = 0.038). These studies show that VR may be an alternative balance intervention for the older adult and PD populations when assessed with TUG outcome measure.

Likewise, improvement with balance outcomes was seen with either the VR treatment group or control group receiving conventional rehabilitation exercises, which further supports that VR intervention can serve as an alternative balance training treatment route to improve balance when assessed with the BBS and TUG in patients with PD [58, 68]. Shih et al. intervened patients with PD with balance-based exergames using a Kinect sensor that encompassed reaching, tracking, obstacle avoidance, and marching tasks [58]. Results related to BBS scores significantly improved from baseline to post-training reported as 50.9 ± 5.32 to 53.2 ± 2.86 for the balance-based exergaming group while the control group receiving conventional balance training also showed significant improvements that were reported as 50.4 ± 4.79 to 53 ± 1.89 from baseline to post-training with a *p* value of 0.001 [58]. In the same study, TUG scores also demonstrated significant improvement from baseline to postintervention with the balance-based exergaming group reported as 9.5 ± 2.45 to 8.71 ± 1, while the control group receiving conventional balance training also significantly improved from 10.05 ± 4.66 to 9.18 ± 3.42 (*p* = 0.007) [58]. Yang et al. [68] also studied patients with PD who were intervened with a custom home-based virtual reality balance training system and reported both the treatment and the control group to have significant changes in BBS, TUG, and Dynamic Gait Index scores within their respective groups from pretest to posttest (*p* < 0.001), but the in-between group differences were not reported as significant. Overall, the above studies support the usage of VR intervention as an alternative balance training intervention for patients with PD, based on improvements in both the BBS and TUG balance outcome measures.

#### 4.3.3. Motor Training following Stroke

Virtual reality has been implemented into treatment for patients following a stroke. The study by Lee et al. studied patient-perceived difficulty using a visual analog scale and the patient's level of enjoyment during the VR activity, as well as the factors that affected them. They found that the factors that affected perceived difficulty and enjoyment include the patient's ease of following directions, their pain experience, scores achieved, novelty and feedback, and self-perceived effectiveness [58]. The authors concluded that implementing VR in a graded manner decreases patient-perceived difficulty and increases patient enjoyment [58]. Another study conducted by Piron et al. used a combination of telerehabilitation and VR in patients with arm motor impairments following a stroke [71]. Telerehabilitation plus VR was compared to traditional therapy, and the results showed that telerehabilitation plus VR demonstrated better outcomes with motor performance [71]. Another study conducted by Lee et al. looked at the use of augmented reality to improve gait function in patients poststroke [65]. The patients in the experimental group received traditional therapy plus augmented reality-based postural control training [65]. The results showed improvements in the TUG, Berg Balance Scale, gait velocity, cadence, step length, and stride length on the patient's paretic side [65]. On the nonparetic side, the patients showed improvements in step length and stride length [65]. These studies demonstrate that the use of a VR-based rehabilitation program may be beneficial to patients who are poststroke.

#### 4.3.4. Use of VR to Address Pain Related to Burns or Phantom Limb

The effects of virtual reality on pain in burn patients and amputees with phantom limb pain have been studied. One study conducted by Khadra et al. [72] studied the feasibility, acceptability, and outcomes of using VR-based therapy to help with pain in children with burn injuries. The children received traditional pharmacological treatment in addition to the VR intervention [72]. The results of the study showed that the children were cooperative and reported low pain scores and low discomfort levels [72]. Soltani et al. [16] studied the effects of VR on pain in patients hospitalized with burn injuries. During physical therapy treatment sessions, the patients performed an active range of motion exercises with and without VR distraction [16]. Participants reported lower scores with VR on the Graphic Rating Scale for worst pain, pain unpleasantness, and amount of time thinking about pain compared to without VR [16]. Joo et al. looked at the use of VR in patients with burned hands. The VR-based rehabilitation was in combination with traditional therapy [73]. The results showed that participants in the VR group showed more improvement on the Jebsen-Taylor hand function test for picking up small objects and the Michigan Hand Outcomes Questionnaire for hand function, functional activities, work, pain, aesthetics, and patient satisfaction compared with the group without VR [73]. The effects, as well as the feasibility, of VR on phantom limb pain in amputees have also been studied. Rutledge et al. [20] looked at phantom limb pain before and after VR treatment. Prior to the treatment, 57% of participants had phantom limb pain and 93% reported phantom sensations that were uncomfortable. Following the VR treatment, only 28% reported phantom limb pain and 28% reported phantom sensations [20]. The participants rated the helpfulness, realism, immersion, and satisfaction of the VR treatment high to very high [20]. This study demonstrated that VR may be a useful intervention for individuals experiencing phantom limb pain.

## 5. Conclusion

In our constantly evolving technological world, the integration of technology in healthcare, particularly in the field of physical therapy, is gaining immense popularity across diverse settings. In this environment, VR stands out as a powerful tool that can be harnessed through various systems, each offering unique characteristics to aid patients in achieving their individualized therapeutic goals.

The application of virtual reality has demonstrated notable benefits for patients struggling with gait and balance disorders, as well as those experiencing burns and phantom limb pain. Furthermore, VR proves to be a valuable assessment tool, comparable to a sensory organization test, enabling the identification of postural deficits and balance impairments in individuals afflicted by Alzheimer's disease and mTBI.

Despite the promising outcomes observed thus far, further research is imperative to dig deeper into the potential advantages of utilizing virtual reality in the realm of physical therapy. By gaining a more profound understanding of VR optimal implementation for patients, we can potentially enhance the efficacy and maximize the benefits of this technology in improving patient outcomes.

## Figures and Tables

**Table 1:** Common VR system used in healthcare.

		Manufacturer	Special characteristics
Virtual reality (VR)	Oculus (Quest 2)	Oculus VR	3D positional audio and 6 degrees of freedom (DFO) sensor to track head movement, provides a higher resolution, and does not require cable connections.
	Oculus (Rift S)	Oculus VR	Rift S provides a software library and easy setup with a PC hardware platform.
	CAREN	Motek Technologies	Includes real-time motion and force capturing system and 6 DOF moving platform and 360-degree theater system.
	Samsung Gear VR	Samsung Electronics Co., Ltd.	Turns a cell phone into a portable VR system and is a lightweight platform to use.
	Psious	Amelia Virtual Care	Focuses on modules for mental health.
	Virtualis	Virtualis VR (Perols, France)	Provides modules for a variety of health conditions to improve activities of daily living (ADL), balance, motor function, hemineglect, cognition, and proprioception.

Augmented reality (AR)	Nintendo Wii variations	Nintendo Co., Ltd.	A VR experience through a player's avatar that captures the overall player's motions with the aid of the handheld device and infrared sensors.
	Kinect for Xbox 360 or Xbox One	Microsoft Corporation	Provides an immersive experience where individuals can use their body to control their game motions.
	Reh@City	Teresa Paulino at Universidade da Madeira	Navigate a city and engage in basic ADLs.
	BTS Nirvana VR	BTS Bioengineering	Provides rehabilitation modules specific for neurological disorders.

## Data Availability

The reference search information data used to support the findings of this study are included within the article.
